# Living Alone, Loneliness, and Psychological Well-Being of Older Persons in Singapore

**DOI:** 10.1155/2011/673181

**Published:** 2011-09-29

**Authors:** Lena L. Lim, Ee-Heok Kua

**Affiliations:** Department of Psychological Medicine, National University of Singapore, Singapore 119074

## Abstract

Studies of the psychological well-being of elderly living alone have yielded inconsistent results. Few investigators have distinguished living alone from loneliness in the same study. Thus, the present study examined the independent and interactive effects of living alone and loneliness on depressive symptoms (GDS score) and quality of life (SF-12 MCS score) in a prospective 2-year follow-up cohort study of 2808 community-dwelling older adults (aged ≥55 years) in Singapore, controlling for baseline covariates. In cross-sectional analysis, loneliness was a more robust predictor of GDS score than living arrangements; living alone, when controlled for loneliness, was not associated with GDS score. GDS score associated with living alone was worse for those who felt lonely than for those who did not feel lonely. Similar patterns of association were found in longitudinal analyses and for SF-12 MCS score, although not all were significant. Thus, though living alone predicted lower psychological well-being, its predictive ability was reduced when loneliness was taken into account and loneliness, a stronger predictor, worsened the psychological effects of living alone.

## 1. Introduction

Population aging generates an array of social and health concerns, among which are the special concerns of the psychological well-being of elderly who live alone. Much research on the association between living arrangements and subjective well-being of the elderly has yielded inconsistent findings. Studies of various older populations in the United States, Hong Kong, Japan, and The Netherlands have reported that elderly living alone were more likely to be depressed [1–5] and have poorer mental health status and quality of life [2, 6, 7] than their counterparts.

However, some authors reported that living alone was not associated with higher levels of depressive symptoms and lower quality of life [3, 8]. For instance, Kawamoto et al. [9] in their 2-year study also found that living alone was not a significant risk factor for emotional well-being in Japanese elderly after adjustment for conventional confounding factors such as age, sex, work activity status, and self-rated health. These discrepant findings may be explained by various reasons that include the selection characteristics and heterogeneity of the study populations, such as differing concentrations of the urban poor and indigent in different studies. An important factor is the effect of loneliness.

Loneliness can be viewed as a subjective measure of one's state of mind and the negative feelings about one's level of social contact [10], often involving an unwanted discrepancy between existing and desired relationships [11]. Loneliness is a universal phenomenon embedded in the human experience and is closely associated with changing life circumstances [12]. Old age is a period that is often seen to be marked by loneliness [13], escalating with approaching death [14]. Studies have shown that loneliness is linked to depression and lower quality of life [8, 15, 16] and increased vulnerability to both physical and mental health problems of the elderly [17, 18]. These associations have been shown to be independent of age, education, income, marital status, and perceived stress [15].

 In much of the earlier literature, the concepts of living alone and loneliness were often used interchangeably [19]. However, living alone is not equivalent to feeling lonely. While living alone is an objective measure of one's living arrangements, loneliness is a subjective emotional experience of one's personal relationships. Hence, although living alone may increase the risk for loneliness, not all elderly people who live alone feel lonely and vice versa.

It has been suggested that the relationship between living alone and psychological well-being may be more salient in Chinese populations [20], in which the collectivist culture places a strong emphasis on family togetherness and the interdependence of family members. Thus, living alone may arguably have a strong negative effect on the well-being of the Asian elderly. As in other developed countries, the population in Singapore is ageing rapidly. The number of elderly aged 65 and above in Singapore has increased markedly from 164,000 in 1990 to 238, 000 in 2000, and it is expected to multiply threefold to 900,000 in 2030 [21]. In this predominantly Chinese population with traditional value of filial piety, children are expected to care for their aging parents; 74% of the elderly live with their families [21]. However, rapidly accelerating population aging and recent sociological trends toward family nuclearization have increased the number of elderly people living alone from 15,000 to 22,000 between 2000 and 2005 [21]. Therefore, in such a highly collectivist society like Singapore [22] which values family unity and interdependence, living alone would have a negative effect on the well-being of the elderly. 

Under the influence of the traditional values such as filial piety which still dominates the family support system of modern collectivist societies including Singapore, adult children are expected to support their parents financially, physically, and emotionally. This traditional cultural practice and attitude enable most elderly to have high level of social engagement and thereby reduce feeling of loneliness. Yang and Victor [23] reported that living in a rural area and thinking one's children as not filial were two significant predictors of old age loneliness that are specific to the Chinese context. The elderly who are most likely to feel lonely would be those who perceived that their adult children had failed to fulfill their responsibilities. Loneliness occurs when there is a difference between the perceived and expected amount of support the elderly derive from their families especially children. 

The present study seeks to examine the unique effects of living arrangements (living alone versus living with others) and loneliness and their interactions on depression and quality of life in a 2-year followup study of community-dwelling elderly in Singapore. We postulated that while living alone might possibly be negatively associated with these measures of psychological well-being, the associated feeling of loneliness might possibly be a stronger contributing factor. Furthermore, the effect of living alone on the psychological well-being might possibly be amplified by the experience of loneliness. These relationships are examined by controlling for the effects of other variables known to influence well-being in the elderly: age, gender, race, marital status, educational level, social contact frequency, number of medical problems, number of social/productive/fitness/health activities, functional disabilities (activities of daily living, ADL), and cognitive status. 

## 2. Methods

### 2.1. Participants

The present study used data drawn from 2808 participants in the Singapore Longitudinal Aging Study (SLAS), a prospective cohort study of aging and health among community-dwelling elderly Singaporeans. All older adults who were citizens or permanent residents aged 55 years or above were identified by door-to-door census and invited to participate voluntarily in the study. The study was approved by National University of Singapore Institutional Review Board. The estimated response rate was 78.5%. 

 Compared to those who dropped out, those who were followed up in the present study included more women, (65.3% versus 58.9%, *P* = 0.001), fewer with low or no educations (23.6% versus 29%, *P* = 0.02), and fewer who were living alone (11.7% versus 15.2%, *P* = 0.01). Those who were followed up gave significantly higher scores on health activities (*P* = 0.01), social activities (*P* = 0.001), and productive activities (*P* < 0.001), higher baseline MMSE score (*P* < 0.001), and lower baseline GDS score (*P* < 0.001), and felt less lonely (*P* = 0.01) than those who dropped out of the study. 

### 2.2. Measurements

Participants underwent an extensive series of health interviews and assessments. Structured interviews, physical performance, and clinical assessments were conducted by trained nurses and clinical psychologists. Interviews were conducted by a multiethnic and multilanguage team in the language or dialect with which the subjects were most conversant with.

**Living Alone**

Participants were asked whether they were currently (1) living alone or with (0) others (spouse, adult children, other relatives, or friends).

**Loneliness**

Participants were asked “Do you feel that at the present moment you are: not at all lonely (= 1), fairly lonely (= 2), very lonely (= 3)?” As there were small numbers of participants who were “very lonely” (and only three participants who were “very lonely” and lived alone), the loneliness variable was dichotomized into “not lonely” and “lonely” (fairly lonely and very lonely).

**Depressive Symptoms**

The 15-item Geriatric Depression Scale [24] was administered as a measure of depression at baseline and at followup. Composite score was calculated based on the unweighted sum of the 15 component items, with a potential range of 0 to 15. Cronbach's alpha in the present study were  .84 and  .79 at baseline and followup, respectively.

**Quality of Life**

The generic health-related quality of life (QOL) was measured with the 12-item Short-Form Health Survey [25]. This shorter version of the commonly used SF-36 yields two summary measures: the physical component summary scale (PCS) and the mental component summary scale (MCS). Summary measures range from 0 to 100 and are calculated using the weighted scores of the twelve items; higher scores represent better QOL. Only MCS was used in this study.

Potential confounders collected at baseline included (1) sociodemographic variables; (2) number of chronic medical problems: participants were asked whether they had been diagnosed and treated by a doctor for a list of medical problem(s) including high blood pressure, high cholesterol, diabetes, stroke, heart attack, atrial fibrillation, heart failure, cataract/major eye problem, kidney failure, asthma, chronic obstructive lung disease, arthritis, hip fracture, and mental illness; the number of chronic medical problems was added for each participant; (3) social contact frequency: frequency of social contract was measured based on the reported frequency of participants' visits and calls by children/relatives/friends, ranging from 3 = at least once a week, 2 = at least once a month, 1 = at least once a year, 0 = none; (4) social activities: participants were asked the number of social activities (in six classes: “Attend church, temple, or mosque,” “Visit cinemas, restaurants, sport events,” “Day or excursive trips,” “play cards, games, bingos, mahjong,” “join a senior citizen club activities,” “Participate in social group activities e.g., karaoke, line dancing”) that they engaged in at least once a month; (5) productive activities: participants were asked the number of productive activities (in six classes: “hobbies, e.g., gardening, painting,” “Preparing meals,” “Shopping,” “Unpaid community work,” “Paid community work,” and “Other paid employment or business”) that they engage in at least once a month; (6) fitness activities: participants were asked the number of fitness activities (“Physical exercises,” “Walking,” “Active sports or swimming,” and “Taiji”) that they engaged in at least once a month; (7) health activities: participants were asked the number of health activities (“Watch what you eat”, “Exercise regularly (i.e., 2-3 times a week),” “Good sleep,” and “Have time for leisure and relaxation”) that they engage in at least once a month; (8) functional status: assessed by the participants' level of dependency in performing 10-item Basic Activities of Daily Living (ADL) found in the Barthel Index [26]; Cronbach's alpha was 0.92 in the present study; (9) cognitive function: assessed by the Mini-Mental State Examination [27], a global measure of cognitive function, which has scores ranging from 0 to 30, with higher scores denoting better cognitive performance. Cronbach's alpha in the present study was 0.83.

## 3. Statistical Analysis

Analysis of covariance (ANCOVA) was used in cross-sectional and longitudinal analyses to examine the relations between baseline values of living alone and loneliness on baseline and followup levels of GDS and SF-12_MCS scores as dependent variables, controlling for covariates including age, gender, race, marital status, educational level, social contact frequency, number of medical problems, number of social/productive/fitness/health activities, baseline levels of depression, activities of daily living (ADL), cognitive function, and mental components of quality of life. Longitudinal analyses included additionally as covariates baseline levels of GDS and SF-12_MCS. *T* test of significance for continuous variables and chi-squared tests of significance for categorical variables were used, with two-tailed significance at *P* < 0.05. SPSS statistical software version 6 (SPSS Inc, Chicago, Ill) was used for all analyses.

## 4. Results

In this population of older adults (mean age 66 years), 211 (7.5%) reported living alone and 344 (11.9%) reported feeling lonely (Table 1).

Seniors who were living alone were more likely to be older, women, non-Chinese, single, divorced or widowed, and without formal education. Notably, they were twice more likely to report feeling lonely (24.2% versus 10.9%). Interestingly, they reported higher frequency of social contact. There were no significant differences in leisure, health and fitness activities scores, number of medical problems, and cognitive and functional disability; but those who lived alone reported significantly higher number of depressive symptoms (2.45 versus 1.85). 

Seniors who were lonely were also more likely to be older, single, divorced or widowed, but did not differ on other socioeconomic characteristics. Similarly, they also reported higher frequency of social contact, but, in contrast, they reported significantly fewer leisure, health and fitness activities, more medical problems, cognitive and functional disability, and depressive symptoms, as well as poorer SF-12_MCS scores. 

The results of the main effects of living arrangements and loneliness and their interactions for depressive symptoms in cross-sectional and longitudinal analyses are shown in Tables 2 and 3, respectively. Controlled for covariates representing important risk and protective factors, living alone was significantly associated with greater GDS score in the cross-sectional analysis (Model 1a). However, this relationship was insignificant when loneliness was included in the model (Model 2). Loneliness, on the other hand, was consistently associated with greater GDS and poorer SF-12_MCS scores, both on its own or together with living arrangements in the model.

Between living alone and loneliness, their respective *R* ^2^ values in the model indicated that loneliness made by far the greater contribution to depressive symptom scores than living alone (see Table 2). For SF-12_MCS scores, both living alone and loneliness, independently of each other, were associated with lower SF-12_MCS scores but did not show significant interaction between them.

 In the cross-sectional analysis, there was a significant interaction between living arrangements and loneliness, F (1, 2536) = 8.28, *P* = 0.004 (Table 2). Consistent with our hypothesis, simple-effects contrast analyses revealed that lonely seniors living alone had higher number of depressive symptoms (M = 5.13, SD = 0.32) than nonlonely seniors living alone (M = 1.16, SD = 0.19), *t* (2567) = 11.1, *P* < 0.001. Lonely seniors living with others (M = 4.17, SD = 0.14) also reported significantly higher number of depressive symptoms than nonlonely seniors living alone, *t* (2567) = 13.9, *P* < 0.001. In longitudinal analyses (Table 3), loneliness was consistently significantly associated with higher GDS and lower SF-12_MCS scores, more than living alone; the combined presence of both was associated with the highest GDS score, although the interaction (*P* = 0.19) was not statistically significant. Finally, using a cutoff score of 5 on the GDS for depression, logistic regression analyses (Table 4) also supported the ANOVA findings (baseline: *χ*² = 339.2, *P* < 0.001; followup: *χ*² = 163.1, *P* < 0.001). The odds ratio for loneliness in predicting depression at baseline and followup was 1.55 and 1.39, respectively, thereby, further underscoring the important role of loneliness on elderly psychological well-being. 

## 5. Discussion

In agreement with previous studies, we found that loneliness was by far a stronger contributor to mental health and functioning. Our study suggests that while living alone contributed to poorer psychological well-being, it was mostly attributed to loneliness. Whether living alone or with others, elderly persons who were not lonely showed similarly fewest numbers of depressive symptoms. However, lonely elderly persons showed higher number of depressive symptoms if they were living alone than if they were living with others. These results from an Asian Singaporean sample are similar to findings from some large surveys in UK and US such as the Health and Retirement Survey, the English Longitudinal Survey, and the Chicago Health and Aging Survey which also used a single-item measure of loneliness. 

These findings help to explain the inconsistent findings reported in previous research on the effects of living alone on depression. Populations of older persons who live alone in different studies may vary in their proportions of those who felt lonely, depending on the ecological context of social support and cohesions. In our study population, this proportion was about a quarter. For example, Mui [5] found that elderly Chinese immigrants in America living alone were more likely to be depressed, but the effect of loneliness was not examined in their study. 

Living alone may predispose to more depression by engendering social isolation especially when social contacts are not maintained [28]. However, this is not invariably the case in all social contexts or communities. It is interesting to note in this study of elderly Singaporeans that those who lived alone in fact reported significantly higher frequency of social contact than their counterparts who lived with others and there was little difference in the levels of social or productive activities between them. This is because elderly who live alone are identified by the Ministry of Community Services for befriender and other social welfare services provided by local resident committees and voluntary organizations. Elsewhere, elderly immigrants who live alone may experience stressors such as language and culture barriers that impede their participation in social activities, hence, intensifying feelings of social isolation and loneliness. 

It is thus interesting to note that, given the traditional preference for Chinese elderly people to live with their adult children especially sons, living alone did not appear to have a profound effect on mental health and functioning as expected. Most probably, the case is that given the same collectivist culture that emphases family togetherness, living alone may not greatly diminish the close knit of family members and their moral obligations for psychosocial support, even though nuclearization of families is on the increase. In Singapore, public policy emphasis and tangible incentives to encourage nuclear families to live in close proximity to elderly parents living alone also help to alleviate the detrimental effects on their psychological and subjective well-being. 

On the other hand, loneliness was found to have a much stronger impact on the psychological well-being of the elderly. The intrinsic emotional bonding between elderly parents and their adult children is more central than physical togetherness in determining mental well-being. We further postulate that the differential effects between living alone and loneliness may lie in the subjective perception of their children as being filial in fulfilling the responsibilities of caring for their parents. Even with children living apart, some elderly may not perceive their needs as unmet by their children, and, hence, there is no negative impact on their well-being. However, those elderly who felt that their children had failed to provide the desired and expected level of support, albeit living with them under the same roof, may be more likely to report poorer mental health and psychological well-being. 

The study has both strengths and limitations. The population-based approach makes the results more widely generalizable. However, special high-risk groups of frail elderly living alone need a separate and more detailed study. In the longitudinal analyses, because the selection characteristics of the participants in the followup interview was clearly biased towards better functioning individuals, the relationships of primary interest were likely to have been attenuated as a result. Self-reported data are subjected to possible social desirability and recall bias, and solely relying on such responses might possibly exaggerate the relationships. The construct of loneliness in this study is simple, and further studies may examine the multiple dimensions of loneliness and their effects on the mental health and functioning of elderly persons.

## Figures and Tables

**Table 1:** Baseline characteristics and well-being of older adults by living arrangement (*N* = 2799).

	Living alone (*N* = 211)				Living with others (*N* = 2588)				*t*	*χ*²	*P*
	Mean	%	SD	*N*	Mean	%	SD	*N*			
Age, years	67.5		7.47		65.9		7.67		2.90		0.004
Gender											
Male		21.3		45		38.0		976		23.3	<0.001
Female		78.7		166		62.0		1592			
Race											
Chinese		91.0		192		93.5		2401		6.60	0.04
Non-Chinese		9.0		19		6.50		167			
Marital status											
Single		28.0		59		4.00		104		455.2	<0.001
Married		16.1		34		78.5		2017			
Divorced/Separated		13.7		29		2.50		61			
Widowed		42.2		89		15.0		385			
Education											
None		28.0		59		18.3		470		14.0	0.007
Primary (1–6 years)		27.5		58		33.6		864			
Secondary (7–10 years)		32.2		68		31.7		814			
Postsecondary		6.60		14		9.70		250			
University		5.70		12		6.70		170			
Loneliness status											
Lonely		24.2		51		10.9		279		36.5	<0.001
Not lonely		75.8		160		89.1		2309			
Social contact score	1.60		0.89		1.36		0.68		4.87		<0.001
Social activities score	3.10		2.29		2.99		2.21		0.68		0.50
Production activities score	4.00		1.78		3.82		1.92		1.34		0.18
Fitness activities score	2.29		1.91		2.52		1.99		−1.60		0.11
Health activities score	5.74		1.81		5.73		1.83		0.03		0.98
No. of medical problems	2.05		1.45		1.98		1.78		0.61		0.54
Baseline ADL	19.9		0.51		19.7		1.48		1.33		0.18
Baseline GDS	2.45		3.39		1.85		2.67		3.07		0.002
Baseline MMSE	26.5		3.53		26.9		3.58		−1.41		0.16
Baseline SF-12_MCS	54.2		7.73		53.7		8.18		0.88		0.38

	Lonely (*N* = 334)				Not lonely (*N* = 2465)				*t*	*χ*²	*P*
	Mean	%	SD	*N*	Mean	%	SD	*N*			

Age, years	67.5		8.64		65.9		7.49		3.40		0.001
Gender											
Male		38.3		128		36.9		830		0.25	0.62
Female		61.7		206		63.1		1419			
Race											
Chinese		90.4		302		93.7		830		5.57	0.06
Non-Chinese		9.60		32		6.30		142			
Marital status											
Single		10.5		35		5.10		115		64.5	<0.001
Married		57.5		192		76.4		1719			
Divorced/Separated		7.50		25		2.40		55			
Widowed		24.5		81		15.9		357			
Education											
None		17.7		59		19.8		445		2.86	0.58
Primary (1–6 years)		35.3		118		32.2		725			
Secondary (7–10 years)		31.7		106		31.5		709			
Postsecondary		8.10		27		9.90		222			
University		7.20		24		6.60		148			
Social contact score	1.61		0.87		1.32		0.65		7.35		<0.001
Social activities score	2.53		1.94		3.10		2.25		−4.39		<0.001
Production activities score	3.30		1.94		3.93		1.89		−5.70		<0.001
Fitness activities score	2.17		1.96		2.57		1.99		−3.44		0.001
Health activities score	5.14		1.98		5.84		1.80		−6.55		<0.001
No. of medical problems	2.20		1.46		1.94		1.80		2.60		0.01
Baseline ADL	19.6		1.73		19.8		1.40		−2.08		0.04
Baseline GDS	4.57		3.99		1.28		1.91		24.5		<0.001
Baseline MMSE	26.2		4.30		27.0		3.41		−3.56		<0.001
Baseline SF-12_MCS	48.6		10.1		54.9		7.09		−7.81		<0.001

**Table 2:** Cross-sectional analysis: associations of living arrangement and loneliness with baseline GDS and SF-12_MCS scores.

Source of variation	Depressive symptoms scores (*N* = 2799)					SF-12_MCS scores (*N* = 2769)				
	df	Mean square	*F*	*P*	*R* ^2^	df	Mean square	*F*	*P*	*R* ^2^
Base model:										
Age	1	63.19	9.06	0.003		1	364.17	5.63	0.02	
Gender (0 = male, 1 = female)	1	3.50	0.50	0.48		1	20.46	0.32	0.54	
Race	1	5.71	0.82	0.37		1	58.43	0.90	0.34	
Education	1	0.15	0.02	0.88		1	7.16	0.11	0.74	
Marital status	1	13.50	1.94	0.16		1	79.10	1.22	0.27	
Number of medical problems	1	202.57	29.06	<0.001		1	861.44	13.32	<0.001	
Baseline ADL	1	24.06	3.45	0.06		1	156.18	2.42	0.12	
Baseline MMSE	1	191.21	27.43	<0.001		1	317.57	4.91	0.03	
Social contact frequency score	1	175.08	25.11	<0.001		1	514.34	7.95	0.005	
Social activities score	1	31.12	4.46	0.04		1	86.30	1.33	0.25	
Production activities score	1	92.58	13.28	<0.001		1	847.28	13.10	<0.001	
Fitness activities score	1	1.45	0.21	0.65		1	198.54	3.07	0.08	
Health activities score	1	313.76	45.00	<0.001	0.081	1	1721.80	26.62	<0.001	0.034
Model 1a: plus living alone versus with others (1,0) only	1	51.85	7.45	0.006	0.083	1	79.03	1.22	0.27	0.034
Model 1b: plus Lonely versus not lonely (1,0) only	1	2414.76	486.86	<0.001	0.231	1	9170.17	165.16	<0.001	0.095
Model 2: plus living alone and loneliness (main effects)										
Living alone versus with others (1,0)	1	2.25	0.45	0.50		1	345.37	6.23	0.01	
Lonely versus not lonely (1,0)	1	2349.77	473.56	<0.001	0.231	1	9294.44	167.59	<0.001	0.096
Model 3: Main effects and interaction										
Living alone versus with others (1,0)	1	19.55	3.95	0.047		1	388.59	7.01	0.01	
Lonely versus not lonely (1,0)	1	1445.06	292.07	<0.001		1	3753.97	67.69	<0.001	
Living arrangements*Loneliness	1	40.85	8.26	0.004	0.232	1	51.08	0.92	0.34	0.096

**Table 3:** Longitudinal analysis: living arrangement and loneliness on psychological well-being at followup.

Source of variation	Depressive symptoms scores at followup (*N* = 1841)					SF-12_MCS scores at followup (*N* = 1790)				
	df	Mean square	*F*	*P*	*R* ^2^	df	Mean square	*F*	*P*	*R* ^2^

Base model:										
Age	1	25.85	9.34	0.02		1	388.21	11.02	0.001	
Gender (0 = male, 1 = female)	1	13.46	4.86	0.03		1	9.74	0.28	0.60	
Race	1	0.44	0.16	0.69		1	1.25	0.04	0.85	
Education	1	2.38	0.86	0.35		1	1.41	0.04	0.84	
Marital status	1	4.40	1.59	0.21		1	9.08	0.26	0.61	
Number of medical problems	1	35.39	12.78	<0.001		1	702.45	19.95	<0.001	
Baseline GDS	1	788.88	284.87	<0.001						
Baseline ADL	1	39.76	14.36	<0.001		1	340.17	99.66	0.002	
Baseline MMSE	1	0.89	0.32	0.57		1	107.09	3.04	0.08	
Social contact frequency score	1	8.40	3.03	0.08		1	121.46	3.45	0.06	
Social activities score	1	21.10	7.62	0.01		1	447.60	12.71	<0.001	
Production activities score	1	35.32	12.75	<0.001		1	156.46	4.44	0.04	
Fitness activities score	1	4.70	1.70	0.19		1	0.06	0.02	0.97	
Health activities score	1	8.74	3.16	0.08	0.220	1	169.03	4.80	0.03	
Baseline SF-12_MCS well-being	—	—	—	—	—	1	4179.08	118.67	<0.001	0.140
Model 1a: plus living alone versus with others (1,0) only	1	5.30	1.91	0.17	0.220	1	130.54	3.71	0.05	0.143
Model 1b: plus Lonely versus not lonely (1,0) only	1	37.67	14.93	<0.001	0.223	1	578.94	17.14	<0.001	0.145
Model 2: plus living alone and loneliness (main effects)										
Living alone versus with others (1,0)	1	4.63	1.84	0.18		1	65.10	1.93	0.17	
Lonely versus not lonely (1,0)	1	35.07	13.91	<0.001	0.221	1	526.48	15.58	<0.001	0.146
Model 3: Main effects and interaction										
Living alone versus with others (1,0)	1	8.56	3.40	0.07		1	152.81	4.53	0.03	
Lonely versus not lonely (1,0)	1	32.57	12.92	<0.001		1	560.42	16.61	<0.001	
Living arrangements*Loneliness	1	4.39	1.74	0.19	0.242	1	110.65	3.28	0.07	0.158

**Table 4:** Logistic regression predicting depression at baseline and followup.

Source of variation	Depression at baseline (*N* = 2799)				Depression at followup (*N* = 1841)			
	B	S.E	Odds ratio	*P*	B	S.E	Odds ratio	*P*
Base model:								
Age	0.03	0.01	1.03	0.004	0.02	0.02	1.03	0.25
Gender (0 = male, 1 = female)	−0.14	0.17	0.87	0.44	−0.33	0.36	0.72	0.35
Race								
Chinese	0.50	0.81	1.64	0.54	−0.58	1.13	0.56	0.61
Malay	−0.51	0.92	0.60	0.58	−0.32	1.28	0.73	0.81
Indian	0.39	0.92	1.48	0.67	−0.85	1.43	0.43	0.55
Education	−0.10	0.08	0.90	0.22	−0.02	0.17	0.98	0.90
Marital status								
Single	0.45	0.33	1.57	0.17	0.86	0.61	2.35	0.16
Married	0.26	0.21	1.29	0.24	0.45	0.40	1.57	0.26
Divorced, separated	0.46	0.39	1.58	0.28	0.64	0.74	1.89	0.39
Number of medical problems	0.17	0.05	1.18	0.001	0.17	0.09	1.18	0.06
Baseline ADL	−0.04	0.04	0.97	0.08	−0.14	0.06	0.87	0.01
Baseline MMSE	−0.09	0.02	0.92	<0.001	−0.002	0.04	1.00	0.96
Social contact frequency score	0.17	0.09	1.18	0.08	0.10	0.18	1.11	0.57
Social activities score	−0.04	0.04	0.96	0.25	−0.11	0.08	0.89	0.16
Production activities score	−0.08	0.05	0.93	0.08	−0.07	0.09	0.97	0.40
Fitness activities score	0.07	0.04	1.07	0.12	−0.04	0.09	0.79	0.70
Health activities score	−0.11	0.04	0.90	0.008	−0.24	0.08	0.93	0.004
Baseline GDS	—	—	—	—	0.29	0.04	1.33	<0.001
Living alone versus with others (1,0)	0.21	0.34	1.23	0.05	0.11	0.71	1.11	0.10
Lonely versus not lonely (1,0)	0.44	0.16	1.55	0.001	0.33	0.36	1.39	0.03
Living arrangements*Loneliness	0.31	0.52	1.36	0.04	0.16	0.33	1.17	0.4
